# Infants at risk for autism: a European perspective on current status, challenges and opportunities

**DOI:** 10.1007/s00787-012-0368-4

**Published:** 2013-01-10

**Authors:** Sven Bölte, Peter B. Marschik, Terje Falck-Ytter, Tony Charman, Herbert Roeyers, Mayada Elsabbagh

**Affiliations:** 1 Center of Neurodevelopmental Disorders (KIND), Department of Women’s and Children’s Health, Karolinska Institutet, Astrid Lindgren Children’s Hospital, Q2:07, 17176 Stockholm, Sweden; 2 Institute of Physiology, Center for Physiological Medicine, Developmental Neurophysiology and Developmental Neuroscience, Medical University of Graz, Graz, Austria; 3 Babylab, Department of Psychology, Uppsala University, Uppsala, Sweden; 4 Department of Psychology, Institute of Psychiatry, King’s College London, London, UK; 5 Department of Experimental Clinical and Health Psychology, Ghent University, Ghent, Belgium; 6 Centre of Brain and Cognitive Development, Birkbeck College, University of London, London, UK; 7 Department of Psychiatry, McGill University, Montreal, Canada

**Keywords:** Autism, Europe, Diagnosis, Technology, Infants, High-risk

## Abstract

Currently, autism cannot be reliably diagnosed before the age of 2 years, which is why longitudinal studies of high-risk populations provide the potential to generate unique knowledge about the development of autism during infancy and toddlerhood prior to symptom onset. Early autism research is an evolving field in child psychiatric science. Key objectives are fine mapping of neurodevelopmental trajectories and identifying biomarkers to improve risk assessment, diagnosis and treatment. ESSEA (Enhancing the Scientific Study of Early Autism) is a COST (European Cooperation in Science and Technology) Action striving to create a European collaboration to enhance the progress of the discovery and treatment of the earliest signs of autism, and to establish European practice guidelines on early identification and intervention by bringing together European expertise from cognitive neuroscience and clinical sciences. The objective of this article is to clarify the state of current European research on at-risk autism research, and to support the understanding of different contexts in which the research is being conducted. We present ESSEA survey data on ongoing European high-risk ASD studies, as well as perceived challenges and opportunities in this field of research. We conclude that although high-risk autism research in Europe faces several challenges, the existence of several key factors (e.g., new and/or large-scale autism grants, availability of new technologies, and involvement of experienced research groups) lead us to expect substantial scientific and clinical developments in Europe in this field during the next few years.

## Introduction

Autism spectrum disorders (ASD) are neurodevelopmental disorders defined by impairments across the areas of reciprocal social interaction, verbal and non-verbal communication, alongside a preference for repetitive, stereotyped activities, patterns of behaviors and interests [3]. Most of the traditional clinical and basic science literature on ASD focused on the phenomenon at the age between 4 and 5 years. Indeed, until some 10 years ago, it was fairly uncommon for children to get diagnosed with autism before the age of 3 or 4 years. Even in today’s clinical practice, in many cases, especially for milder variants of ASD, late ASD diagnoses frequently occur. This is despite the fact that both major diagnostic systems (DSM-IV-TR, ICD-10) in their editions published in the early to mid-90s defined an early onset of symptoms (<36 months of age) as essential for classical autism. In addition, the concept of ASD in general implies pervasive developmental delay and/or deviance in a multitude of basic functions, such as play, motor development, attention, adaptive behavior, particularly social reciprocity and verbal as well as nonverbal communication being apparent from early childhood onwards. Also, most parents are concerned about their children’s behavior from early on. In a study by Chawarska et al. [5], the average age of first parental concerns was 14 months. On the other hand, there is a substantial minority of autistic individuals who develop typically or apparently typically at first (up to 24 months of age), but later show a loss of skills and developmental regression [15]. Furthermore, the symptom severity in ASD is variable, as are socio-communicative, speech-language and intellectual skills, and children with ASD might present with striking co-existing problems unspecific to ASD (e.g., irritability, hyperactivity, sleep and feeding problems) or may appear typically developing to the non-experienced or untrained observer. Hence, early ASD detection in infants remains challenging.

In recent years, a growing interest in infant development and early detection of ASD has emerged, mostly driven by the insight that early identification is a prerequisite for early intervention, which itself may improve long-term outcomes for individuals with ASD [6]. Several methodologies have helped to study early detection and examine early development in ASD. Screening instruments for early signs of ASD, such as the CHecklist for Autism in Toddlers (CHAT), Early Screening for Autistic Traits (ESAT), the Modified-CHAT, and the Infant Toddler Checklist (ITC) have demonstrated the possibility to prospectively identify ASD at 18 months or even earlier (for reviews see [1, 4]) in low-risk and high-risk populations. Common early signs are primarily delays and deficits in response to name and joint attention and limited or perseverative early play. Nevertheless, many of these signs are neither specific for nor universal to ASD, with low positive predictive values, and a risk for overreferral particularly in case of one stage screening. Another method to study early development in ASD is retrospective analyses of home videos collected prior to diagnosis. Palomo, Belinchón, and Ozonoff [19] summarized eight such studies from the first 2 years of life of children who were later diagnosed with ASD. Consistent early signs in the first year of life were reduced response to name as well as reduced frequency of looking at faces. During the second year of life pointing to request and to show, as well as showing/giving objects were the most prominent atypical features.

To focus on the early development and early detection of autism, an alternative and increasingly applied approach is the longitudinal study of infant siblings of children with ASD. Infant siblings are at increased risk (~20 %) of developing ASD compared to 1 % of the general population, with the risk being higher for males than for females, and higher for those from multiplex (>1 sibling) then simplex families [18]. By monitoring developmental trajectories in high-risk siblings more precise information about the first appearance of autistic behaviors has evolved (for reviews, see [20, 25, 27]). A note of caution is that it is a matter of debate whether ASD aetiologies are comparable between simplex and multiplex families with ASD [14], so there may be limits on the extent to which findings from high-risk infant sibling studies generalize to the simplex population. High-risk studies consistently reported that while infants later diagnosed with ASD exhibit signs of the condition at 12 months (e.g., lack of eye contact, reciprocal smiling, and social engagement), no such behavioral differences have been reliably detected at 6 months [17]. Nevertheless, the search for behavioral and biological markers with sufficient sensitivity and specificity to be of clinical feasibility and validity using a multitude of techniques [e.g., questionnaires, behavioral observation, eye tracking, magnetic resonance imaging (MRI), event related potentials (ERPs)] in high-risk autism populations is an ongoing quest. Most recent evidence from eye tracking studies, including those from ESSEA laboratories (Enhancing the Scientific Study of Early Autism), suggests that subtle joint attention difficulties at 13 months might be related to ASD or other altered neurodevelopmental outcomes [2], and that children with poor social and communicative skills differ from typically developed concerning eye gaze patterns leading to successful word learning [12]. On the other hand, in a recent US study decreased eye contact in high-risk siblings at 6 months was not related at all to ASD outcome at 24 months [16, 26]. Parent-infant interaction observations in 6- to 10-month-old infants found less liveliness in the at-risk sample, and more directedness as well as lower sensitive responsiveness of their parents, compared to low-risk controls [21]. Interestingly, two independent studies [22, 23] suggest that some of the earliest risk markers may lie within the motor domain. With respect to electrophysiological evidence, Elsabbagh et al. [8], using ERPs found that response to dynamic eye gaze shifts during the first year (6–10 months of age) was associated with ASD diagnosed at 36 months. Other neuroimaging work from the US infant brain imaging study (IBIS) network using diffusion tensor imaging found aberrant white matter fiber tract at 6 months and subsequent tract trajectories to be associated with and ASD diagnosis at 24 months [24].

Generally, enhanced collaboration and networking are crucial in moving forward with challenging scientific questions in early autism research. There is a need for sharing protocols and data between labs to advance practice and influence policy makers. While consortia like the IBIS and Baby Sib Research Consortium (BSRC) are ongoing in North America, until recently a comparable and competitive coherent Europe-wide network has not yet been established. ESSEA (for details please see http://www.cost-essea.com/) is a COST (European Cooperation in Science and Technology) action striving to establish an interdisciplinary scientific network to advance the pace of discovery about the earliest signs of autism; to combine techniques from cognitive neuroscience with those from the clinical sciences; and to generate European practice guidelines on early identification and intervention. ESSEA is funded under the EU’s Seventh Framework Programme for Research (FP7) via the European Science Foundation (2011–2014). It is a network of over 60 scientists from 22 European countries and various scientific disciplines. COST is a means for European researchers to jointly develop new ideas and initiatives across scientific disciplines through trans-European networking of nationally funded research activities. COST provides financial support only for joint activities such as conferences, short-term scientific exchanges, training schools and publications. ESSEA intends to develop European capacity in early autism research. The lack of a forum to enhance the scientific synergies between these strands of basic and applied research has previously hindered progress. Increased and earlier recognition has impacted across Europe in terms of demand for diagnostic services and interventions. Current health care systems across Europe are very variable in terms of their expertise and capacity to support families with young children with autism, often leading to marginalization. Although the primary focus of ESSEA is research, it offers the potential to help build capacity for health systems and clinical care (e.g., raise awareness, spread expertise, provide research to practice solutions, and propose evidence-based clinical guidelines).

The goal of this article is to review European research capacities in the field of at-risk for ASD research and to support the understanding of differences in European research contexts. The findings might be valuable to overcome research impediments, and to highlight how using specific strengths of the European collaborative sites might be used to this effect, as well as to raise awareness and influence public policy toward greater engagement in ASD issues in Europe. For this purpose, two surveys were sent to members of ESSEA actively involved in high-risk research and/or in the development of novel methods for early autism research (WG1 and WG2 members): first, one on ongoing full scale, pilot, as well as planned high-risk studies in Europe among ESSEA sites; and second, another on perceived challenges and opportunities for autism research in general and high-risk research in particular among those European research sites.

## Methods

### Instruments

#### High-risk studies and applied technologies

For the survey of ESSEA members’ ongoing or planned high-risk autism studies, and applied technologies, ESSEA member principal investigators completed an open item format investigator questionnaire. It inquired about ongoing or planned research projects, methodologies, technologies, collaborative attitudes, priorities, collaborators, interests, funding, and relevant publications in early autism research. For researchers currently or soon to start conducting research with populations at risk for autism (investigators involved in collaborative projects to complete the survey jointly) the survey required information on the status, principal investigators, project title, current sample size, project’s total sample size, key words for focus of study, type of risk sample, methodologies and technologies employed, and project start.

#### Barriers and opportunities

For the mapping of existing, experienced, and perceived circumstances that may hamper (“barriers”) or may strengthen (“opportunities”) progress in research and cooperation in the area of autism in general and high-risk studies in particular on a national and European basis a 25-item questionnaire using a closed format was developed. It explores funding, recruitment, awareness, traditions, cooperation with public and interest organizations, ethics, and the availability of instruments/methods, and qualified personnel. It contains 20 questions on barriers and 5 on opportunities (see Table 1). For each barrier or challenge item respondents needed to quantify, whether the specific issue inquired about posed no, a mild, a moderate, or severe barrier. For each opportunity, respondents evaluated whether the specified issue had no, minor good, or excellent potential within their research environment.

**Table 1:** Summary of ESSEA locations, enrollment numbers, and methodologies

Status	Country	Principal Investigator(s)	Project title	Current *N*	Total *N*	Topic key words	Risk Groups	Methodology/technologies	Project start
Ongoing full-scale	UK	M. H. Johnson	British Autism Study of Infant Siblings (BASIS)-UK research network www.basisnetwork.org	188	260	Brain, social, attention, motor, BAP, genetics, intervention	Infant ASD siblings	Genetics, EEG, ERP, MRI, DTI, NIRS, behavior, eye tracking, standardized measures, questionnaires	2008
	Belgium	H. Roeyers	Social cognitive mechanisms of understanding intentional agents in typical and atypical development	60	150	Social development, attention	Infant ASD siblings	EEG, ERP, behavior, eye tracking, standardized measures, questionnaires	2007
				0	100		Preterm infants		2012
	Sweden	U. Ådén B. Vollmer S. Bölte	Follow up of extremely preterm infants at 6 years—a population-based study in Stockholm (NeoBIC)	50	117	Brain, cognition	Preterm infants	Standardized measures, questionnaire, MRI	2004
	Sweden	S. Bölte T. Falck-Ytter G. Gredebäck	Early Autism Sweden (EASE) www.earlyautism.se	30	100	Non-verbal communication, BAP, autonomous system, motor function, brain, microbiota	Infant ASD siblings	Eye tracking, MRI, DTI, EEG/ERP, behavior, standardized measures questionnaires	2011
				0	30		Infants with fetal alcohol/neonatal abstinence syndrome		*TBD*
	Italy	T. Farroni	Predictors at birth and in premature babies	3	*TBD*	ASD predictors	Preterm infants	NIRS, behavioral	2010
Pilot	Czech Republic	M. Hrdlicka	New approaches to early diagnostics of autism	5	12	Genetics	ASD siblings	Karyotyping, screening, CNV analysis	2011
	Israel	N. Yirmiya	Association between preterm birth and ASD spectrum disorders and the BAP	25	100	ASD phenotype	Preterm infants	Observation, interview	2009
	Israel	D. Mankuta	Prenatal clues for Autism in high-risk groups	35	300	Biochemical, sonographic, genetic markers	Infant ASD siblings	Ultrasound, amniotic fluid analyses	2012
Planned	Portugal	A. Vicente	Earliest signs of autism	0	200	Genetics	*TBD*	CGH arrays, questionnaires	2012
	Spain	M. Posada R. Canal	Spanish ASD sibling cohort: feasibility study	0	*TBD*	Feasibility, epidemiology	Infant ASD siblings	Screening	2011

*TBD* to be determined, *BAP* broader autism phenotype

### Participants, procedure and analyses

Eighteen sites from 17 European countries completed the initial mapping of studies and technologies between January and March 2011: UK, Ireland, Hungary, Belgium, Spain, Sweden, The Netherlands, Finland, France, Norway, Romania, Italy, Macedonia, Czech Republic, Portugal, Germany, and Israel (2 sites). The surveys were sent via e-mail to the principal investigators or research groups’ contact persons, and returned electronically. The returned material was analyzed, and summarized to form an overview on studies and technologies with high-risk autism research. Countries without ongoing or planned high-risk studies were excluded. Finally, the overview was sent to the informants for approval. Before submission (August 2012) of this article some numbers were updated in this overview, particularly the current *N* of enrolled participants.

With regards to the mapping exercise on barriers and opportunities 16 sites from 15 countries responded between October 2011 and February 2012: UK, Ireland, Belgium, Spain, Sweden, The Netherlands, France, Romania, Italy, Macedonia (2 sites), Czech Republic, Portugal, Austria, Germany, and Israel. In cases where clarification was needed to complete the questionnaire, these issues were discussed during the regular WGs telephone conferences, or via e-mail. Questionnaire data were entered in SPSS 19, and analyzed descriptively on item level for frequency distribution of scores. To avoid bias towards one specific European country and because the pattern of responses were similar, the data from the two Macedonian sites was collapsed to one.

## Results

### High-risk studies and applied technologies

An overview of the relevant projects is given in Table 2. In four countries full scale studies on high-risk autism populations are currently conducted: in the UK, the Baby Sibling Study network (BASIS; http://basisnetwork.org) headed by the Centre for Brain and Cognitive Development at Birkbeck University; in Belgium, at Ghent University, Department of Experimental Clinical and Health Psychology, Research group Developmental Disorders (Baby Study; http://ontwikkelingsstoornissen.ugent.be/babystudie); in Sweden, the Early Autism Sweden Study (EASE, http://earlyautism.se) at Karolinska Institutet, Department of Women’s and Children’s Health (KIND) and Uppsala University, Department of Psychology (Babylab); as well as in Italy, at Padua University. Participants also reported that pilot and planned studies are ongoing in the Czech Republic, Israel, Portugal, and Spain. A substantial body of literature on the high-risk autism field is already available from some of these groups (e.g., [7–13]). Populations examined are in the majority autism siblings, but also preterm babies. The Swedish site also aims to include children with fetal alcohol syndrome and neonatal abstinence syndrome. The target N for at-risk siblings/preterm infants of the studies varies between 12 and 300, with the BASIS network currently having enrolled most high-risk children and low-risk controls (*N* ~ 200). Applied assessments range from behavior and neurobiology/neurophysiology to genetics. Among the technologies applied in infants are (quantitative) EEG, ERP, eye tracking, functional near-infrared spectroscopy (fNIRS), structural MRI, functional MRI, diffusion tensor imaging, copy number variants analysis, and comparative genomic hybridization-arrays.

**Table 2:** Survey results for mapping research barriers (items 1–20) and opportunities (items 21–25) among ESSEA members

	No barrier (%)	Mild barrier (%)	Moderate barrier (%)	Severe barrier (%)
1. There are funding challenges for high-risk autism research	26.7	13.3	60.0	0
2. There are funding challenges for autism research in general	20.0	20.0	60.0	0
3. There are general research funding challenges	26.7	13.3	33.3	26.7
4. There are general challenges in recruiting any high-risk sample (e.g., sibs, preterms)	26.7	13.3	60.0	0
5. There are challenges in recruiting large sample sizes of high-risk subjects*	21.4	0	42.9	35.7
6. Parents are negative/uncooperative towards autism research in general**	92.3	0	0	7.7
7. Parents are negative/uncooperative towards high-risk autism research	93.3	0	0	6.7
8. Parent-/interest organizations are negative/uncooperative towards autism research in general	93.3	0	0	6.7
9. Parent-/interest organizations are negative/uncooperative towards high-risk autism research	86.6	0	6.7	6.7
10. There are challenges in cooperating with/getting support from public (private) organizations (nursery, school, health care etc.) for autism research in general*	35.7	28.6	28.6	7.1
11. There are challenges in cooperating with/getting support from public (private) organizations (nursery, school, health care, etc.) for high-risk autism research*	43.0	14.2	35.7	7.1
12. Autism is stigmatized*	57.2	21.4	14.3	7.1
13. Autism awareness is low	46.7	33.3	20.0	0
14. There exists an anti-psychiatry sentiment*	50.0	21.4	14.3	14.3
15. There are certain traditions (psychoanalytic, psychosocial, “humanistic”) that hamper autism research	53.3	33.3	6.7	6.7
16. Our country is not perceived as developed enough to carry-out research on high-risk autism populations	93.3	0	0	6.7
17. There are challenges in obtaining diagnostic and research tools (e.g., tests, software, apparatus), due to several factors (no adaptions available, no shipping, no administrative rights to use, etc.)	60.0	13.3	20.0	6.7
18. There are difficulties in recruiting adequate personnel (e.g., no interest, no education, place is too expensive for academic salaries, insufficient English language skills)	60.0	13.3	26.7	0
19. There are challenges in obtaining ethical permission for high-risk research*	57.2	28.6	7.1	7.1
20. There are other autism research barriers, particularly with regards to high-risk research, that have not been addressed in the survey**	76.9	15.4	7.7	0
21. There are funding opportunities for autism research in general and high-risk autism studies in particular***	33.3	41.7	16.7	8.3
22. There are sample recruitment opportunities for autism research in general and high-risk autism studies in particular**	30.8	30.8	38.4	0
23. There are parent/interest organization opportunities for autism research in general and high-risk autism studies in particular**	15.4	23.1	53.8	7.7
24. There are other societal opportunities (e. g., high awareness, trust in research, public interest) for autism research in general and high-risk autism studies in particular**	30.8	46.1	23.1	0
25. There are opportunities with regards to methods, personnel, and ethics for autism research in general and high-risk autism studies in particular***	50.0	8.3	41.7	0

* *N* = 14, ** *N* = 13, *** *N* = 12

### Barriers and opportunities

Detailed findings are shown in Table 2. Almost three-quarters of the responding sites report mild to moderate barriers in the areas of funding high-risk research and recruiting adequate samples and sample sizes. More than 60 percent mentioned mild to severe problems when contacting and trying to collaborate with public organizations (e.g., preschool, health care). Stigmatization surrounding autism was perceived as a mild to severe problem in over 40 % of the responding countries, while in only one country lacking cooperation of interest and parent organizations was perceived as a severe challenge. Autism awareness was judged to be low by almost half of the countries. A more general anti-psychiatry sentiment, and certain traditions (“psychosocial”, “humanistic”, “psychoanalytic”) were also experienced to moderately or severely hamper autism research by half of the respondents. In addition, 40 % or more of respondents reported challenges concerning the availability of adequate diagnostic instruments, qualified personnel, or getting ethical approval. Finally, more than 20 % reported other challenges not explicitly covered by the survey, such as interdisciplinary cooperation (e.g., with obstetrics and neonatology), conducting longitudinal research, educational prerequisites, and more specific issues with regard to the standardization and validation of psychometric tools. When members of participating ESSEA countries were asked about strengths and possibilities for autism and high-risk research, >20 % reported good to excellent funding, 30–40 % good to excellent recruitment and personnel, and >50 % good to excellent collaboration with interest organizations.

## Discussion

Researchers from 17 different European countries contributed to our surveys, suggesting that our results are fairly representative of the situation in Europe regarding research on autism early in life. Our surveys identified a number of unique strengths. Studies on high-risk for autism populations and the use of novel technologies are evolving in Europe. Despite the relatively small number of studies, current projects range from large-scale collaborative initiatives to small pilots, and planned projects. Current high-risk populations under investigation are infant siblings of children with ASD, preterm infants, as well as individuals with neonatal abstinence syndrome and fetal alcohol syndrome. Areas of expertise include genetics, neurolinguistics, neuroscience, developmental science and clinical research. Applied technologies are equally broad, and include eye tracking, ERP/EEG, MRI, and fNIRS. Particularly with the BASIS network in the UK, Europe has established an internationally recognized cohort study, and other groups are catching up. Moreover, about 20–50 % of the respondents reported moderate to excellent opportunities for research funding, recruitment of infants, employment of adequately educated personnel, and cooperation with parent, and interest organizations. Finally, initiatives such as COST facilitate interaction between sites through specific mechanisms such as lab exchanges, training schools, enhanced communication, etc. Against this background, there seems to be a good potential to further develop at-risk research and collaboration in Europe to advance the pace of change in basic and clinical science relative to early autism in the coming years.

We also identified substantial challenges for high-risk autism research and usage of technologies in Europe. Aside from funding and recruitment problems in some countries, a majority mentioned limited support by public organizations (e.g., preschool, health care). Also, stigmatization of children with autism, low autism awareness, anti-psychiatry opinions, limited availability of adequate diagnostic instruments and qualified personnel, or getting ethical approval as well as predominating traditions with low scientific or empirical background still prevent a better situation for high-risk research in many parts of Europe. Information on these impediments may serve as a starting point for additional in-depth examination of such challenges, as well as to inform national and European organizations with an interest in autism and autism research and health care policy makers. Moreover, the ESSEA network may help to eliminate these barriers, through active dissemination of the present results on the situation in Europe to the public, generating proposed European guidelines for ethical management, and develop standards and recommendations for responsible and effective communication of scientific evidence and opportunities.

ESSEA is a potentially powerful initiative to improve the situation for early autism research in Europe. In order to enhance the prerequisites for a large-scale research platform in Europe, that can compare for instance with the primarily US-based BSRC (http://autismspeaks.org/science/initiatives/high-risk-baby-sibs) or IBIS (http://ibis-network.org) networks, ESSEA will as next steps among other things: explore the feasibilities of a data repository for common standardized measures, standardization of ethical and clinical management approaches of European high-risk studies, and identify and agree on common measures. Further support and synergies for high-risk autism science in Europe can be expected from another European autism research collaboration, the innovative medical initiatives project EU-AIMS (www.eu-aims.eu): European autism interventions—a multicentre study for developing new medications. This consortium project includes multiple European sites conducting high-risk infant studies amongst many other basic science and clinically relevant tasks (clinical infrastructure; outcome measures; education and training; biomarkers) that overlap both with regards to objectives and academic centers with ESSEA.

An increasing interest and progress in the field of high-risk autism research and usage of technology in Europe is apparent. Nevertheless, despite a few pioneer research groups, Europe cannot yet compare to the level of experience, expertise, networking and funding achieved in North America. With the ESSEA COST action and the EU-AIMS IMI consortium project the European Union has funded initiatives that promise to make European research on early autism internationally competitive. The next years will be decisive for European autism scientists to convince European funders that this money is well spent, and to motivate continued support and funding.
